# Recent Advances in the Application of VO_2_ for Electrochemical Energy Storage

**DOI:** 10.3390/nano15151167

**Published:** 2025-07-28

**Authors:** Yuxin He, Xinyu Gao, Jiaming Liu, Junxin Zhou, Jiayu Wang, Dan Li, Sha Zhao, Wei Feng

**Affiliations:** College of Chemistry, Chemical Engineering and Resource Utilization, Northeast Forestry University, Harbin 150040, China

**Keywords:** vanadium dioxide, lithium-ion batteries, zinc-ion batteries, photoassisted batteries, supercapacitors

## Abstract

Energy storage technology is crucial for addressing the intermittency of renewable energy sources and plays a key role in power systems and electronic devices. In the field of energy storage systems, multivalent vanadium-based oxides have attracted widespread attention. Among these, vanadium dioxide (VO_2_) is distinguished by its key advantages, including high theoretical capacity, low cost, and strong structural designability. The diverse crystalline structures and plentiful natural reserves of VO_2_ offer a favorable foundation for facilitating charge transfer and regulating storage behavior during energy storage processes. This mini review provides an overview of the latest progress in VO_2_-based materials for energy storage applications, specifically highlighting their roles in lithium-ion batteries, zinc-ion batteries, photoassisted batteries, and supercapacitors. Particular attention is given to their electrochemical properties, structural integrity, and prospects for development. Additionally, it explores future development directions to offer theoretical insights and strategic guidance for ongoing research and industrial application of VO_2_.

## 1. Introduction

Energy is a fundamental driving force of societal development and serves as the material foundation of daily human life. Energy storage devices not only absorb surplus energy to prevent waste but also release it during critical periods, thereby ensuring the safe and efficient use of energy. In modern society, electricity has penetrated all aspects of daily life, ranging from lighting and household appliances to transportation. Batteries and capacitors play indispensable roles in electronic devices and power systems. Batteries provide high energy density for sustained operation [1], whereas supercapacitors deliver rapid charge/discharge capabilities to enable instantaneous power delivery [2]. Collectively, they form fundamental components of modern electronics and energy infrastructure.

To design high-performance energy storage devices, a variety of functional materials have been developed, including transition metal oxides (e.g., VO_2_, V_2_O_5_ [3,4], MnO_2_ [5,6,7]) and carbon-based materials (e.g., graphene [8], carbon nanotubes [9,10]), MXenes [11], and transition metal chalcogenides (e.g., MoS_2_ [12]). These materials exhibit excellent electrochemical properties, tunable structures, and strong photoelectric responses, and have been widely employed in high-capacity, high-efficiency, and long-lifespan lithium-ion batteries, zinc-ion batteries, and other energy storage systems.

Among these materials, VO_2_, a representative vanadium-based oxide, has attracted considerable attention because of its high theoretical capacity and excellent chemical stability. Owing to its diverse crystalline phases and abundant availability, VO_2_ can efficiently regulate charge transport and storage during electrochemical cycling, demonstrating great potential in the development of multifunctional and high-performance energy storage systems. This paper systematically reviews recent advances in VO_2_-based materials for energy storage technologies, with a particular focus on their applications in lithium-ion batteries, zinc-ion batteries, photoassisted batteries, and supercapacitors. The review is based primarily on high-quality publications from the past three years, aiming to capture the most up-to-date and impactful progress in the field. It also discusses future research directions, providing a theoretical foundation for ongoing and future developments.

## 2. Crystalline Structure and Synthesis of VO_2_

### 2.1. Crystalline Structure of VO_2_

VO_2_ has a variety of crystalline phases, making it a promising candidate for diverse applications. It exists in several polymorphic forms, including the tetragonal rutile phase VO_2_ (R), the monoclinic phase VO_2_ (M), the triclinic phase VO_2_ (T), and several metastable phases, such as VO_2_ (A), VO_2_ (B), VO_2_ (C), VO_2_ (D), VO_2_ (P), and VO_2_ (N) [13,14,15,16,17,18,19,20,21]. This review highlights four common VO_2_ polymorphs: VO_2_ (B) [17], VO_2_ (D) [19], VO_2_ (M) [14], and VO_2_ (R) [13].

**Figure 1:**
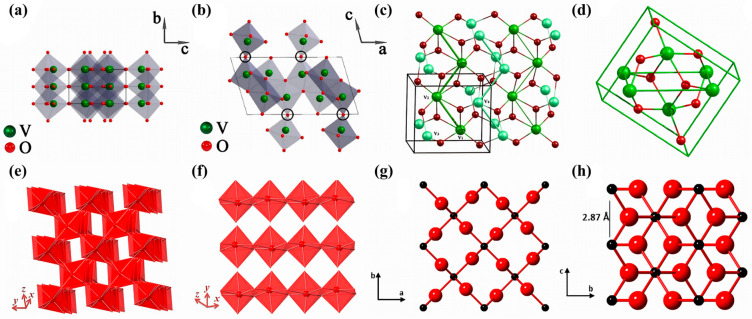
Structure of monoclinic VO_2_ (B): observed along the (**a**) a-axis and (**b**) b-axis. Reproduced with permission [17]. Copyright 2022, MDPI. (**c**) Structure of VO_2_ (D) with large and small balls representing V and O ions, respectively. (**d**) Unit cell of VO_2_ (D). Reproduced with permission [19]. Copyright 2011, Elsevier. (**e**,**f**) Schematic geometries of the crystal structure of monoclinic VO_2_ (M). Reproduced with permission [14]. Copyright 2020, Elsevier. (**g**) Crystal structure of VO_2_ (R) along the tunnel axis. The black and red spheres represent V and O atoms, respectively. (**h**) Crystal structure of VO_2_ (R) along the lateral axis. Reproduced with permission [13]. Copyright 2018, American Chemical Society.

VO_2_ (B) is a metastable monoclinic phase with lattice parameters of a = 12.09 Å, b = 3.702 Å, c = 6.433 Å, and β = 106.6°, and it belongs to the space group C 2/m. The VO_2_ (B) lattice comprises two layers of distorted VO_6_ octahedra aligned along the b-axis (Figure 1a,b). Each vanadium atom is situated at the center of an octahedron and is surrounded by six oxygen atoms, forming VO_6_ units. These [VO_6_] octahedra form double chains and are connected into a two-dimensional layered structure via corner-sharing oxygen atoms [17]. The structure is characterized by interlayer voids and tunnels, which provide effective channels for ion intercalation and deintercalation. Moreover, this architecture can effectively mitigate volume expansion during electrochemical cycling.

VO_2_ (D) is another metastable monoclinic phase with space group P 2/c and lattice parameters of a = 4.60 Å, b = 5.68 Å, c = 4.91 Å, and β = 89.39° [19]. Its structure comprises [V_(1)_O_6_] octahedra that share edges to form zigzag chains (Figure 1c,d). These chains are interconnected with those formed by [V_(2)_O_6_] octahedra through corner-sharing oxygen atoms, resulting in a three-dimensional framework.

VO_2_ (M) is classified as a monoclinic phase with space group P2_1_/c and lattice parameters a = 5.75 Å, b = 4.53 Å, c = 5.38 Å, α = γ = 90°, and β = 122.60°. In this configuration, vanadium atoms form alternating zigzag chains along the *x*-axis (Figure 1e,f). These chains display alternating V–V bond lengths of 2.65 Å and 3.12 Å, indicating the occurrence of V–V dimerization [14]. The structure consists of twisted VO_6_ octahedra connected through coplanar edges, and the chains are further bridged by corner-sharing oxygen atoms to form a robust three-dimensional framework.

VO_2_ (R) crystallizes in a tetragonal rutile structure with a space group of P4_2_/mnm and lattice parameters a = b = 4.55 Å, c = 2.88 Å, and β = 90° [13]. In this structure, V^4+^ ions occupy the corners and body center of the unit cell and are coordinated by six O^2−^ ions to form nearly regular [VO_6_] octahedra (Figure 1g,h). These octahedra are connected via edge sharing along the c-axis, forming continuous linear chains. Between adjacent chains, the octahedra are linked through corner sharing, resulting in a highly symmetric and thermally stable metallic framework.

In summary, each VO_2_ polymorph exhibits distinct structural characteristics that influence its suitability for energy storage [22,23]. VO_2_ (B), with its open-layered monoclinic structure and interconnected tunnels, offers fast ion diffusion and high specific capacity, making it highly promising for battery and supercapacitor applications. VO_2_ (D) shares similar diffusion pathways and electrochemical activities but has been less studied due to phase instability and synthesis challenges. VO_2_ (M), the thermodynamically stable phase at room temperature, provides good structural integrity and moderate performance but suffers from a dense framework that limits rate capability. VO_2_ (R), despite its high electronic conductivity and symmetrical rutile structure, is thermally unstable under ambient conditions and readily reverts to VO_2_ (M), thus limiting its practical applicability. These differences underscore the importance of phase selection and structural tailoring in optimizing VO_2_-based materials for energy storage devices.

### 2.2. Synthetic Method of VO_2_

In the field of electrochemical energy storage, VO_2_ has gained significant attention because of its excellent ion mobility and high specific capacity. The development and optimization of its synthesis methods have remained key areas of focus for both academia and industry. This mini review briefly outlines the main synthesis strategies for VO_2_ used in energy storage applications, including hydrothermal synthesis, pyrolysis, electrodeposition, pulsed laser deposition (PLD), and other methods.

#### 2.2.1. Hydrothermal Methods

The hydrothermal method is one of the most widely used techniques for the synthesis of VO_2_ materials. It involves a dissolution–crystallization process in an aqueous solution under high-temperature and high-pressure conditions. For VO_2_ hydrothermal synthesis, pentavalent vanadium sources such as V_2_O_5_ and ammonium metavanadate are commonly employed, whereas oxalic acid and formic acid serve as reducing agents. By adjusting the synthesis parameters, such as the reaction temperature, duration, and reducing agent type, nanomaterials of various sizes and morphologies, including nanosheets, nanorods, nanoflowers, and nanoparticles, can be obtained.

**Figure 2:**
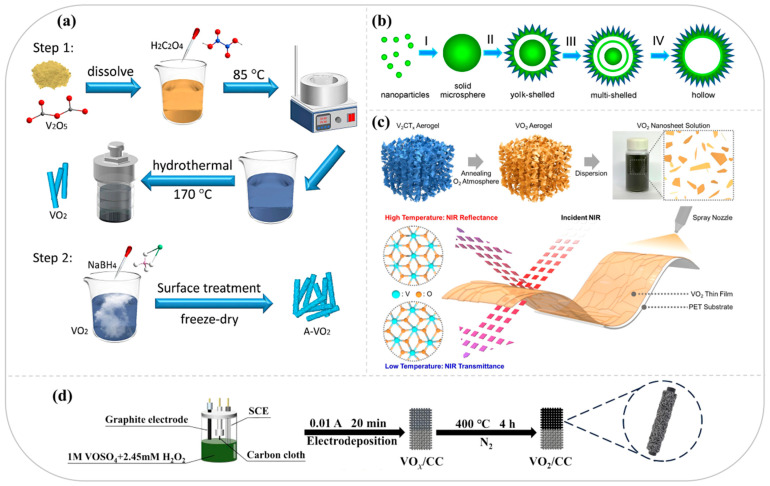
(**a**) Schematic diagram of the synthesis process of VO_2_ and A-VO_2_ samples. Reproduced with permission [24]. Copyright 2024, Elsevier. (**b**) Time-dependent structural evolution of VO_2_ microspheres. Reproduced with permission [25]. Copyright 2013, Wiley-VCH GmbH. (**c**) Schematic for the fabrication of V_2_CT_x_-nanosheet-converted VO_2_ nanosheets. Reproduced with permission [28]. Copyright 2023, Elsevier. (**d**) Illustration of the synthetic procedure of VO_2_/CC. Reproduced with permission [30]. Copyright 2024, Elsevier.

Hou et al. synthesized homogeneous VO_2_ (B) nanorods from V_2_O_5_ and oxalic acid via a facile and rapid hydrothermal method [24]. A heterogeneous structure (A-VO_2_) with a crystalline core and an amorphous shell layer was further constructed by disorder–order engineering of VO_2_ (B) nanorods through a simple reduction treatment in NaBH_4_ solution (Figure 2a). The tunable amorphous layer introduces abundant oxygen vacancies, thereby facilitating ion and electron transport and enhancing structural stability. Pan et al. successfully synthesized VO_2_ (D) hollow microspheres with yolk-shell, multishell, and monoshell structures via a one-step template-free solvothermal approach [25]. The internal structure can be controlled by adjusting the reaction time and precursor concentration (Figure 2b). The hollow structure remained intact after calcination, and the resulting VO_2_ (D) hollow composite structure exhibited high stability, mitigating the agglomeration and dissolution issues typical of conventional solid particles and thereby enhancing ion transport. Liu et al. synthesized nsutite-type VO_2_ microcrystals composed of nanosheets using NH_4_VO_3_ as the precursor and thioacetamide as the reductant [21]. These microcrystals were then assembled into zinc batteries as cathode materials. The microcrystalline structure collapsed into nanosheets during discharge and reassembled into nanosheets during charging. Furthermore, the nanosheets were converted into nanoplates approximately 100 nm thick during charging, and this reversible structural change significantly improved the cycling stability and ion transport efficiency of the material.

The hydrothermal method operates under mild conditions with precise control over temperature and pressure, enabling high crystallinity, uniform particle size, and controlled morphology. However, it typically involves long reaction times and is less suitable for large-scale production.

#### 2.2.2. Pyrolysis Methods

Pyrolysis refers to the synthesis of VO_2_ nanostructures by exploiting the thermal instability of precursors such as vanadates, vanadium-based oxides, and their hydrates, which are decomposed through thermal treatment in an anaerobic environment at medium to high temperatures. Thakur et al. successfully prepared VO_2_ (M) nanopowders with particle sizes < 30 nm through pyrolytic decomposition of the precursor [NH_4_]_5_[(VO)_6_(CO_3_)_4_(OH)_9_]·10H_2_O [26]. The significant release of gases such as NH_3_ and CO_2_ during pyrolysis promotes the nanosizing of VO_2_ (M) powders. By adjusting the pyrolysis conditions, such as the precursor size, heating rate, and gas flow rate, the stoichiometric ratio (VO_2 ± x_, x = 0.04–0.07) and crystalline structure (monocrystalline, nanocrystalline, and amorphous) of the products can be tailored.

Jung et al. developed a method to synthesize high-purity monoclinic phase VO_2_ (M) powders via a mild pyrolysis process without an inert gas atmosphere. VO_2_ (M) powder can be synthesized within 1 h at a low temperature [27]. Thermal analysis revealed that the pyrolysis temperature should not exceed 253 °C to prevent oxidation to V_2_O_5_. The precursor vanadium ethanolate was synthesized and heat-treated for 1 h at 190 °C with an airflow rate of 10 L min^−1^, resulting in VO_2_ (M) powders consisting of 20–50 nm spherical nanoparticles that agglomerated into porous nanorods. These nanorods exhibit reversible metal–insulator phase transition properties.

Pyrolysis allows stoichiometric control through thermal decomposition and is scalable for large-scale production. However, high temperatures may cause particle agglomeration and limit morphology control.

#### 2.2.3. Other Methods

The electrodeposition method allows precise control over the morphology and thickness of the deposited layer by regulating the current density and time. It can be directly deposited on complex substrates without the need for additional binders, making it suitable for the development of energy storage electrodes. Lai et al. used a constant-current electrodeposition method to prepare amorphous VO_4_ nanowires from VOSO_4_ as a vanadium source, with H_2_O_2_ as the oxidizing agent, on carbon cloth [28]. These nanowires were subsequently transformed into uniform, porous VO_2_ (B) nanowires by annealing and crystallization (Figure 2d). This VO_2_ (B) phase has many active sites and fast ion diffusion capabilities, leading to high performance in zinc-ion memory devices. However, this VO_2_ (B) phase can irreversibly convert to V_2_O_5_·H_2_O, causing capacity degradation. To address this, structural design modifications and other approaches are necessary to inhibit phase transition and vanadium solubilization, thereby increasing cycling stability. Xiang et al. developed a simple method for synthesizing high-quality VO_2_ (M) thin films on polymer substrates by preparing VO_2_ (M) films through room-temperature PLD followed by annealing at 390 °C in an oxygen atmosphere [29]. The resulting VO_2_ (M) films exhibit excellent electrical phase transition properties and outstanding optical modulation. By coupling tungsten doping with film strain, the transition temperature of VO_2_ (M) can be adjusted to room temperature with a doping concentration of only 1.1% while maintaining a high electrical contrast of two orders of magnitude. PLDs allow for precise structural engineering and direct integration with substrates, although their scalability and cost-effectiveness remain limited. Kim et al. proposed a facile and scalable strategy for the first successful synthesis of two-dimensional VO_2_ (M) nanosheets with a high aspect ratio and high crystallinity through the heat treatment of monolayer V_2_CT_x_ MXene nanosheets (Figure 2c). The resulting VO_2_ (M) nanosheets can be directly sprayed onto flexible or nonplanar substrates to form dense and uniform films that exhibit excellent thermochromic properties [30].

In addition, sputtering [31] and electrostatic spinning [32] methods have also been employed for the fabrication of VO_2_ nanostructures. These techniques offer distinct advantages in regulating nanostructure morphology (e.g., nanowires, nanofilms, and nanofibers) and crystallinity, providing diverse approaches for the controllable preparation of VO_2_ functional materials. Overall, each method has unique trade-offs in terms of structural control, energy efficiency, and compatibility with device fabrication.

Despite these advancements, many current synthesis routes still face challenges related to high energy consumption, complex processing steps, and limited scalability. Addressing these limitations will require the development of simplified, low-temperature, and continuous synthesis strategies. Solid-state reactions with optimized precursors and hydrothermal processes using recyclable solvents and shorter reaction times are especially promising for facilitating industrial-scale production.

In parallel, VO_2_ itself has relatively low toxicity and good chemical stability under typical operating conditions, supporting its potential use in both large-scale and wearable energy storage systems [33,34]. Nonetheless, despite the effective suppression of vanadium ion leaching through surface modifications [35], further comprehensive studies on its long-term biocompatibility and environmental impact remain essential to ensure safe and sustainable use.

## 3. Application of VO_2_ in Electrochemical Energy Storage

Among the various materials explored for energy storage applications, VO_2_ has attracted considerable attention because of its moderate cost, structural tunability, and well-rounded electrochemical performance (Table 1). Compared with other vanadium oxides (e.g., V_2_O_5_, V_6_O_13_) and emerging materials such as MXenes (e.g., Ti_3_C_2_I_2_) and MoS_2_, VO_2_ offers a more balanced profile in terms of capacity, stability, scalability, and cost [36,37,38,39,40,41]. While V_2_O_5_ and V_6_O_13_ provide low cost or high capacity, they suffer from poor cycling stability. Ti_3_C_2_I_2_ has excellent conductivity but lacks sufficient capacity and scalability, and MoS_2_ offers good stability with relatively low energy density. These trade-offs highlight VO_2_ as a competitive and practical candidate for next-generation rechargeable batteries.

In the following sections, recent advances in VO_2_-based materials over the past three years are systematically reviewed, with a focus on their applications in lithium-ion batteries, zinc-ion batteries, photoassisted batteries, and supercapacitors.

### 3.1. Lithium-Ion Batteries

Among various emerging energy storage technologies, lithium-ion batteries are widely regarded as the dominant option because of their high energy density, long cycle life, and well-established industrial infrastructure [42,43]. In recent years, VO_2_ has attracted considerable attention as a cathode material for high-capacity lithium-ion batteries owing to its unique layered structure and reversible lithium storage capability.

**Figure 3:**
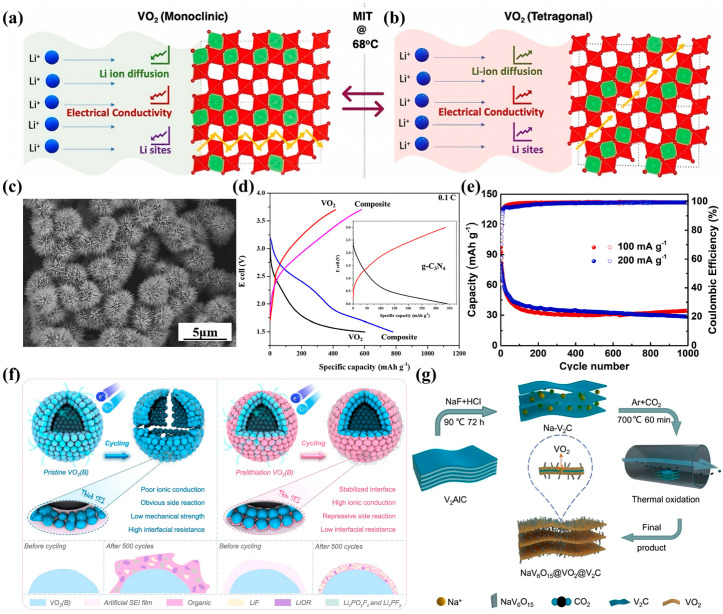
Simulated crystal structure evolution during Li insertion: (**a**) VO_2_ (M); (**b**) VO_2_ (R). Reproduced with permission [44]. Copyright 2024, Royal Society of Chemistry. (**c**) SEM images of flower-like VO_2_ (B) microspheres. Reproduced with permission [45]. Copyright 2024, Elsevier. (**d**) GCD profiles of the VO_2_, g-C_3_N_4_, and composite electrodes at a 0.1 C current density. Reproduced with permission [46]. Copyright 2023, Elsevier. (**e**) Cycling performance of the VO_2_ (D)/LiMn_2_O_4_ full cell at 100 and 200 mA g^−1^. Reproduced with permission [48]. Copyright 2022, Elsevier. (**f**) Schematic of the VO_2_ (B) prelithiation process and its structural advantages over pristine VO_2_ (B). Reproduced with permission [47]. Copyright 2024, Elsevier. (**g**) Schematic illustration of the preparation of the NaV_6_O_15_@VO_2_@V_2_C composite. Reproduced with permission [49]. Copyright 2023, Elsevier.

Castro-Pardo et al. systematically investigated the influence of the metal–insulator transition (MIT) of VO_2_ on its electrochemical performance as a lithium-ion battery cathode [44]. Their findings demonstrated that transitioning VO_2_ from the monoclinic (M) phase to the rutile (R) phase near 68 °C led to an ~70% increase in specific capacity, significantly enhanced rate performance, and reduced charge-transfer resistance. These improvements originate from a series of structural and electronic changes during the MIT. In the low-temperature monoclinic phase, lithium ions migrate through zigzag diffusion paths formed by distorted V–V chains, which impose high migration energy barriers. Upon transition to the rutile phase, the V–V chains straighten into a linear arrangement, forming direct, low-resistance channels for Li^+^ diffusion (Figure 3a,b). This structural reconfiguration significantly lowers the diffusion barrier, as evidenced by a tenfold increase in the Li^+^ diffusion coefficient. Simultaneously, the MIT drastically enhances the electronic conductivity by 3–5 orders of magnitude, ensuring better charge transport throughout the electrode. DFT calculations further show that the rutile phase accommodates a higher lithium filling fraction (0.67 vs. 0.42 per VO_2_ unit) and offers more energetically favorable insertion sites, facilitating a higher reversible capacity. Together, these synergistic effects of structural alignment and electronic enhancement make the VO_2_ (R) phase more suitable for fast and stable lithium storage. In summary, the MIT in VO_2_ provides a unique mechanism to dynamically optimize both ionic and electronic transport, thereby improving the capacity, rate capability, and overall electrochemical performance under thermal activation.

Flower-like VO_2_ (B) microspheres self-assembled from ultrathin nanosheets were synthesized by Liang et al. (Figure 3c). The resulting three-dimensional nanostructures exhibited a specific surface area of up to 30.05 m^2^ g^−1^, which significantly enhanced the lithium-ion transport kinetics [45]. When used as a cathode material, the initial discharge specific capacity reached 209.6 mAh g^−1^ at a high current density of 1 A g^−1^, with a capacity retention of 83.1% after 200 cycles. This excellent performance was attributed to the synergistic effects of the three-dimensional structure in shortening the ion diffusion pathways, buffering volume expansion, and enhancing the electrode–electrolyte interface. In addition, heterostructures were constructed by Jang et al. to effectively integrate the performance advantages of multiple materials. A one-dimensional/two-dimensional (1D/2D) heterostructure composed of VO_2_ (B) nanowires and g-C_3_N_4_ nanosheets [46] was synthesized via a hydrothermal method (Figure 3d). The resulting composite exhibited a high specific capacity of up to 779 mAh g^−1^ at a current density of 0.1 A g^−1^, significantly outperforming pure VO_2_ (601.4 mAh g^−1^). This enhanced performance was attributed to the high electrical conductivity of g-C_3_N_4_, the rapid ion transport provided by VO_2_ (B) nanowires, and their synergistic effect in improving structural stability, which effectively mitigated damage caused by volumetric changes during cycling.

VO_2_ has also been extensively investigated as an anode material for lithium-ion batteries; however, the low initial coulombic efficiency (ICE) and unstable solid electrolyte interphase (SEI) during cycling remain major obstacles to its practical application. To address this challenge, a nondestructive chemical prelithiation strategy was proposed by Yan et al. to significantly improve the ICE of VO_2_ (B) (approaching 100%) and enhance both its cycling stability and overall electrochemical performance [47]. Chemical prelithiation using a lithium aromatic reagent produced a thinner and more uniform SEI film enriched with highly conductive LiF components, which effectively suppressed side reactions (Figure 3f). The prelithiated VO_2_ (B) electrode retained a high reversible capacity of 375 mAh g^−1^ after 1000 cycles at a current density of 1.0 A g^−1^. The application of a VO_2_ (D) submicron spherical hierarchical structure as an anode material for aqueous lithium-ion batteries was first reported by Ma et al. [48]. The material was synthesized via a template-free solvothermal method and demonstrated good structural stability and electrochemical performance. The constructed VO_2_ (D)/LiMn_2_O_4_ full cell delivered a high specific capacity of up to 97.43 mAh g^−1^ across a wide voltage window (0.2–1.8 V). Mechanistic analysis indicated that the unique three-dimensional structure of VO_2_ (D) facilitates ion and electron transport, which expands the application prospects of polycrystalline VO_2_ (D) in energy storage systems (Figure 3e).

VO_2_-based electrodes are prone to failure because of structural degradation during cycling. Repeated ion insertion and extraction cause lattice strain and microcracks, compromising the reversibility of the material. A NaV_6_O_15_@VO_2_ (M)@V_2_C composite with a hierarchical two-dimensional architecture was developed by Tan et al., in which VO_2_ (M) nanosheets are surrounded by NaV_6_O_15_ nanorods and embedded in a dual-conductive network of V_2_C MXenes [49], effectively enhancing the lithium-ion storage capacity and structural stability (Figure 3g). When applied as an anode in lithium-ion batteries, the material achieved a reversible capacity of 408.1 mAh g^−1^ after 100 cycles at a current density of 100 mA g^−1^ and retained 204.5 mAh g^−1^ after 400 cycles at 1 A g^−1^, with a coulombic efficiency of 99.63%. The conductive network not only accelerates lithium-ion migration but also plays a crucial role in mitigating structural collapse by effectively alleviating the stress induced by volume changes during cycling. This modification reinforces structural stability, thereby enhancing the durability and performance of the electrode.

### 3.2. Zinc-Ion Batteries

Zinc-ion batteries are among several battery systems that are adaptable to both aqueous and nonaqueous electrolytes. Compared with other energy storage technologies, these systems have garnered significant attention because of their high safety, environmental friendliness, and low cost. Unlike lithium-ion systems, ZIBs operate in neutral or mildly acidic aqueous electrolytes, which present new challenges for electrode materials, particularly with respect to structural stability, Zn^2+^ diffusion kinetics, and redox reversibility. The unique layered and tunneled crystal structure, multivalent transition capability, and rapid ion transport characteristics of VO_2_ make it a promising candidate for Zn^2+^ storage.

**Figure 4:**
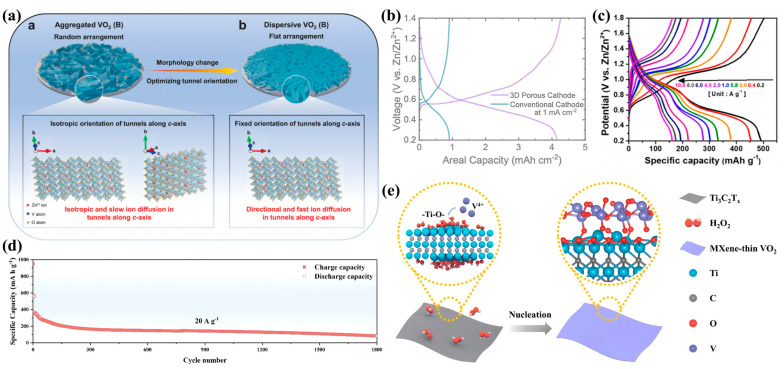
(**a**) Schematic illustration of the effect of the material morphology-related electrode arrangement on the tunnel orientation and ion diffusion behavior. Reproduced with permission [50]. Copyright 2020, Wiley-VCH GmbH. (**b**) GCD curve comparison of 3D and conventional cathodes at 1 mA cm^−2^. Reproduced with permission [51]. Copyright 2025, Royal Society of Chemistry. (**c**) GCD curves of pp-fibers@VO_2_ (B) at current densities from 0.2 to 10.0 A g^−1^. Reproduced with permission [52]. Copyright 2024, Wiley-VCH GmbH. (**d**) Long-term cycling of the D-VO_2_ cathode. Reproduced with permission [53]. Copyright 2025, Elsevier. (**e**) Schematic diagram of the predicted formation mechanism of the heterointerface. Reproduced with permission [54]. Copyright 2025, Royal Society of Chemistry.

He et al. modulated the material morphology to optimize the tunneling orientation in electrodes, and (00l) facet-dominated VO_2_ (B) nanobelts with dispersion (VO_2_-D) were fabricated. These electrodes exhibited excellent rate performance and cycling stability due to their c-axis-oriented tunneling structure (Figure 4a). A specific capacity of 420.8 mAh g^−1^ at 0.1 A g^−1^ and 344.8 mAh g^−1^ at 10 A g^−1^ was achieved, with a capacity retention of 84.3% after 5000 cycles. This offers a novel strategy to enhance ion transport kinetics in tunneling vanadium oxides through the concurrent modulation of exposed crystal facets and morphology-dependent electrode architectures [50]. Pinnock et al. enhanced the performance of aqueous ZIBs by increasing the specific capacity of VO_2_ (B) from 310 to 500 mAh g^−1^ through the optimization of hydrothermal synthesis and freeze–drying treatment. The optimized cathode achieved a stable capacity retention of 71.5% after 1000 cycles [51]. Furthermore, ultrahigh-loading three-dimensional electrodes with a mass loading of up to 24 mg cm^−2^ were fabricated by depositing VO_2_ (B) onto porous glassy carbon foam (Figure 4b), achieving an area capacitance of 4.15 mAh cm^−2^ at 1 mA cm^−2^ and maintaining 81.5% capacity retention after 1000 cycles. These improvements were attributed to the large surface area and excellent ion permeability provided by the 3D structure, which effectively alleviated performance degradation under high-loading conditions. Yeon et al. developed a binder-free VO_2_ composite electrode utilizing polydopamine-derived pyrolytic protein fibers (pp-fibers) as a flexible current collector, enabling enhanced flexibility and electrochemical stability [52]. The binder and additive-free system was synthesized via the hydrothermal growth of VO_2_ (B) nanosheets on pp-fibers. The electrode demonstrated outstanding electrochemical properties in aqueous ZIBs, delivering a specific capacity of 491 mAh g^−1^ at 0.2 A g^−1^ (Figure 4c) and a minimal capacity decay rate of 0.001% per cycle over 20,000 cycles at 1 A g^−1^. Moreover, the assembled flexible pouch cells remained operational under mechanical deformation, offering a promising strategy for the development of flexible energy storage devices.

Vanadium vacancies were introduced into tunneled VO_2_ via hydrothermal synthesis and chemical etching, enabling the modulation of the vanadium valence state and lattice contraction to improve structural stability [53]. These vacancies induce the formation of high-valence vanadium ions, alter the surface charge distribution, and introduce abundant electrochemically active sites that increase the capacity. Furthermore, ion diffusion and electron transport are facilitated through the reduction of the Zn^2+^ migration barrier and charge transfer activation energy. The modified VO_2_ cathode maintained a capacity of 332 mAh g^−1^ after 200 cycles at 0.1 A g^−1^, demonstrating superior cycling stability at low current densities (Figure 4d).

Although oxygen vacancies (O_v_) can expand the lattice and promote Zn^2+^ intercalation, they are prone to being filled by oxygen from the electrolyte and may contribute to capacity degradation due to localized electric field migration. While heterostructure interfaces have shown the ability to modulate the electronic structure of _Ovs_, effective strategies to improve their stability remain underdeveloped. Fang et al. constructed covalent heterostructures featuring Ti-O-V asymmetric orbital hybridization by growing VO_2_ nanowalls on MXene nanosheets via a H_2_O_2_-assisted hydrothermal process (MXene-VO_2−x_) [54]. This interfacial orbital hybridization promoted electron transfer from VO_2_ to the MXene, thereby stabilizing the oxygen vacancies both thermodynamically and kinetically (Figure 4e). A reversible capacity of 487.9 mAh g^−1^ at 0.2 A g^−1^ and a retention rate of 98.6% after 30,000 cycles at 20 A g^−1^ were achieved. Moreover, the flexible devices remained functional under mechanical deformation, offering a novel orbital engineering approach for the rational design of highly reversible ZIB cathodes.

Current strategies to increase the performance of VO_2_-based zinc-ion batteries involve multidimensional approaches, including elemental doping, structural modulation, and heterostructure construction. Table 2 summarizes recent VO_2_-based cathode materials along with their test conditions and electrochemical performance metrics, serving as a valuable reference for ongoing and future research in this field.

As shown in Table 2, VO_2_-based cathodes clearly exhibit trade-offs between capacity, stability, and structural complexity. High-performance composites such as VO_2_/V_2_C@CNF and MXene-VO_2−x_ deliver outstanding specific capacities (up to ~550 mAh g^−1^) and excellent cycling stability (over 85% retention for thousands of cycles) [54,55], primarily due to enhanced electronic conductivity and reinforced structural integrity from carbonaceous frameworks. However, these systems often involve complex synthesis processes, which may hinder large-scale application. In contrast, simpler VO_2_ phases such as VO_2_ (B) offer more accessible fabrication routes but typically suffer from lower long-term stability and capacity fading. Surface functionalization strategies (e.g., H-VO_2_@GO) effectively buffer volume changes and improve ion transport, resulting in significantly enhanced capacity retention [56]. Defect engineering, as in O_v_-CoVO, also contributes to improved reaction kinetics and exceptional stability (~97% retention) [57]. Moreover, some materials emphasize specific performance metrics at the expense of others. For example, D-VO_2_ has a high initial capacity but poor retention at high rates [53], whereas VO@NDA sacrifices the capacity to achieve an ultralong cycling life [58].

These examples collectively underscore that while various modification strategies can greatly enhance VO_2_ cathode performance, they often entail trade-offs among energy density, rate capability, structural stability, and scalability. Therefore, a rational design must balance these factors to meet the demands of practical zinc-ion battery applications.

**Table 2:** Recent progress in the use of VO_2_ and its modified materials as cathode materials for zinc-ion batteries.

Materials	Cell Type	Voltage Range (V)	Capacity (mA h g^−1^)	Capacity Retention (Cycles)	Ref.
VO_2_-D	half-cell	0.2–1.5	420.8 (0.1 A g^−1^)	84.3 % (5 A g^−1^, 5000)	[50]
VO_2_ (B)	half-cell	0.2–1.6	447 (0.2 A g^−1^)	71.3 % (2 A g^−1^, 1000)	[51]
D-VO_2_	half-cell	0.2–1.6	332 (0.1 A g^−1^)	46.2 % (20 A g^−1^, 1800)	[53]
VO_2_	half-cell	0.3–1.3	317 (0.5 A g^−1^)	81.0 % (10 A g^−1^, 2000)	[59]
H-VO_2_@GO	half-cell	0.2–1.5	400.1 (0.5 A g^−1^)	96.1 % (10 A g^−1^, 1500)	[56]
pp-fibers@VO_2_ (B)	half-cell	0.2–1.8	491 (0.2 A g^−1^)	80.17 % (1 A g^−1^, 20,000)	[52]
CrVO	half-cell	0.2–1.3	312.8 (0.1 A g^−1^)	90.39 % (57 A g^−1^, 2000)	[60]
O_v_-CoVO	half-cell	0.3–1.4	475 (0.2 A g^−1^)	97.5 % (5 A g^−1^, 3000)	[57]
MnVO	half-cell	0.3–1.4	209.6 (0.1 A g^−1^)	80.7 % (5 A g^−1^, 10,000)	[61]
Mg-VO_2_	half-cell	0.2–1.5	385.7 (0.1 A g^−1^)	70.5 % (2 A g^−1^, 800)	[62]
Ce-VO_2_	half-cell	0.2–1.4	371.4 (0.1 A g^−1^)	85.0 % (5 A g^−1^, 2000)	[63]
O_v_-VO_2_@CNF	half-cell	0.2–1.4	450 (0.1 A g^−1^)	85.0 % (5 A g^−1^, 2000)	[64]
V_2_O_3_/VO_2_@S/N-C	half-cell	0.2–1.6	257.8 (1 A g^−1^)	81.8 % (200 A g^−1^, 150,000)	[65]
V_6_O_13_/VO_2_	half-cell	0.2–1.6	498.3 (0.2 A g^−1^)	96.8 % (10 A g^−1^, 5000)	[66]
VO_2_/V_2_C@CNF	half-cell	0.2–1.7	549 (0.1 A g^−1^)	87.0 % (10 A g^−1^, 5000)	[55]
MXene-VO_2−x_	half-cell	0.2–1.6	487.9 (0.2 A g^−1^)	98.6 % (20 A g^−1^, 30,000)	[54]
Mo-VO_2_	half-cell	0.4–1.5	409.3 (0.1 A g^−1^)	89.5 % (2 A g^−1^, 1000)	[67]
VO@NDA	half-cell	0.4–1.6	241 (10 A g^−1^)	97.45 % (10 A g^−1^, 15,000)	[58]

Note. Abbreviations used: “CrVO” refers to Cr-ion-doped VO_2_ (B); “O_v_-CoVO” represents oxygen-deficient Cosubstituted VO_2_; and “MnVO” indicates Mn-ion-doped VO_2_.

### 3.3. Photoassisted Batteries

Although solar energy is one of the cleanest and most abundant energy sources, its development and utilization still require significant improvement. Photoassisted batteries, as an emerging technological system that integrates light energy conversion with electrochemical energy storage, exhibit distinct technical advantages through the synergistic optimization of light excitation and battery performance. Its core mechanism involves the use of semiconductors, quantum dots, and other photoactive materials to generate photogenerated carriers under illumination, which promote electrochemical reactions at both electrodes through interfacial charge transfer or directly convert light energy into chemical energy for storage.

Previous studies have demonstrated that VO_2_ has notable electrochemical advantages in lithium and zinc battery systems, with its multiple valence states and reversible phase transitions offering an ideal platform for ion storage. Owing to its dual functionalities in ion storage and light absorption, the coupling of the semiconductor characteristics of VO_2_ with its ion intercalation properties presents significant potential for research on light-induced charge injection and storage mechanisms. Extensive research has been conducted on the application of VO_2_ in photoassisted batteries [68,69], which not only broadens its functional scope but also offers novel theoretical insights for the development of photoelectrochemical synergistic multienergy storage systems.

**Figure 5:**
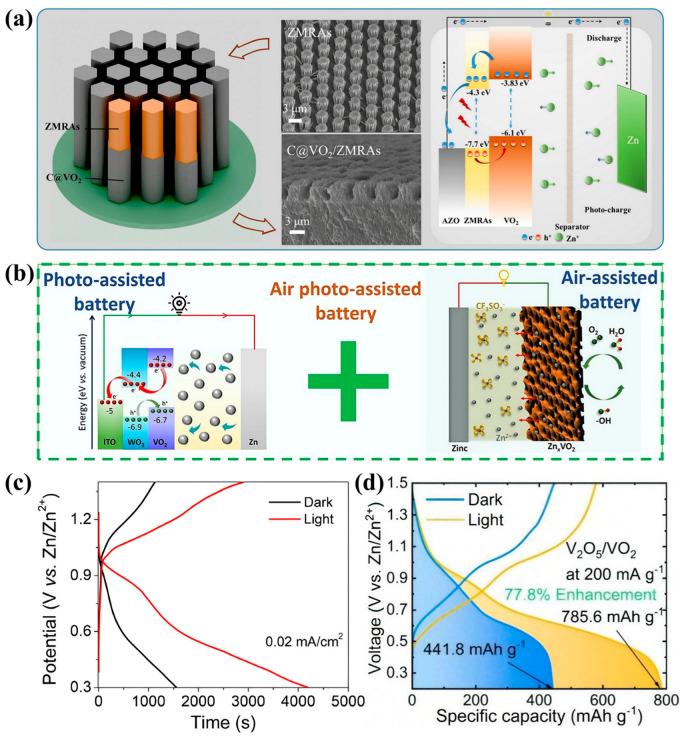
(**a**) Schematic of the preparation process for vertically ordered ZMRAs. Reproduced with permission [70]. Copyright 2024, Elsevier. (**b**) Demonstration of self-rechargeable energy storage using sustainable sources such as solar and air. (**c**) GCD curves of VO_2_/WO_3_ under both dark and light conditions. Reproduced with permission [71]. Copyright 2024, Elsevier. (**d**) Discharge curves of ZIBs with V_2_O_5_/VO_2_ photocathodes under dark and light conditions at 1000 mA g^−1^. Reproduced with permission [72]. Copyright 2025, Royal Society of Chemistry.

Recently, numerous innovative studies in the field of photoassisted batteries have emerged. Ding et al. fabricated a highly ordered, vertically aligned C@VO_2_/ZnO microrod array by combining photolithography with a hydrothermal method to synthesize precursor ZMRAs [70], which were then composited with carbon-coated VO_2_ to construct a three-dimensional heterojunction network (Figure 5a). The heterostructure facilitates the separation of photogenerated carriers, while the carbon coating improves the electrical conductivity, and the 3D network increases the specific surface area, providing additional Zn^2+^ storage sites. This synergistic interaction among components resulted in an 18.6% increase in battery capacity under light illumination. The resulting photorechargeable zinc-ion battery was capable of direct light charging without the need for external photovoltaic modules, achieving a photoconversion efficiency of 3.3%. It delivered a capacity of 286.0 mAh g^−1^ in the dark at a current density of 500 mA g^−1^, which increased to 339.3 mAh g^−1^ under illumination, with a capacity retention rate of 88.79% after 300 cycles at 1000 mA g^−1^. Roy et al. were the first to develop an air-assisted, self-charging energy storage device by employing WO_3_ as a charge-separating layer in combination with VO_2_ to form a heterostructure (Figure 5b). In this system, VO_2_/WO_3_ serves as the cathode, where the cubic WO_3_ structure (200–300 nm) is integrated with the micrometer-scale lamellar VO_2_, thereby increasing the specific surface area of the electrode and optimizing the ion diffusion pathways. Additionally, the high electrical conductivity of WO_3_ reduces the charge-transfer resistance and enhances the electrochemical activity. The built-in electric field at the heterojunction interface significantly enhances the separation of photogenerated charges [71]. The battery capacity increases by 170% under light exposure at a current density of 0.02 mA cm^−2^, and the open-circuit voltage reaches 0.9 V within 140 s during air-assisted self-charging (Figure 5c). Yang et al. synthesized V_2_O_5_/VO_2_ hollow nanorods as cathode materials for photocharged zinc-ion batteries via controlled oxidation [72]. In this system, uniquely structured WO_3_/VO_3_ hollow nanorods were also employed as cathode materials. The unique heterojunction architecture and hollow morphology enable multiple synergistic effects. The built-in electric field of the type-II heterojunction accelerates the transfer of photogenerated electrons from VO_2_ to V_2_O_5_, whereas the holes remaining in VO_2_ facilitate Zn^2+^ deintercalation. The hollow structure enhances light absorption by increasing the specific surface area, resulting in a surface photovoltage of 2573 mV. This design achieved a light-specific capacity of 785.6 mAh g^−1^ at a current density of 200 mA g^−1^ (Figure 5d), representing a 77.8% increase compared with the dark state. A photoconversion efficiency of 4.3% was recorded, with 53.1% capacity retention after 4000 cycles at 1000 mA g^−1^, demonstrating excellent long-term stability.

Although photoassisted batteries currently exhibit relatively low photoconversion efficiencies and are unable to match the performance of conventional PV systems, they present a highly promising avenue for future energy technologies. Their integrated nature offers unique advantages in terms of system simplification and space efficiency. Continued efforts in material innovation and system-level optimization are essential to unlock their full potential and bring them closer to practical deployment.

### 3.4. Other Batteries and Supercapacitors

The potential of VO_2_ extends far beyond current applications, as it has also exhibited a promising performance in emerging energy storage systems such as calcium-ion batteries (CIBs), lithium–sulfur batteries, aluminum-ion batteries (AIBs), and supercapacitors. Its polycrystalline structure, reversible multivalent redox behavior, wide electrochemical window, and excellent structural stability make VO_2_ suitable for diverse ion storage mechanisms. For example, its layered or tunneled structure enables the reversible intercalation of large-radius ions such as Ca^2+^ and Al^3+^; in Li-S batteries, it serves as a polar host to suppress the shuttle effect of lithium polysulfides (LiPS); and in supercapacitors, it delivers outstanding pseudocapacitive performance.

**Figure 6:**
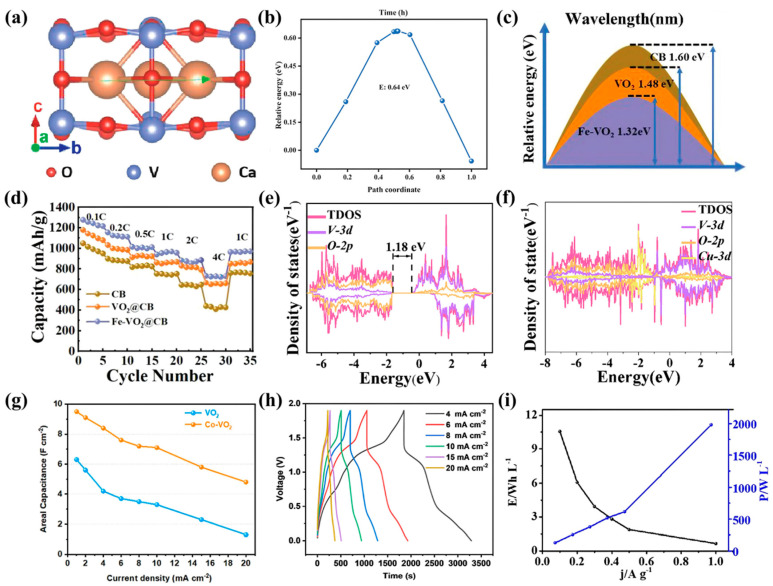
(**a**) Diffusion path distribution of Ca^2+^ in VO_2_ (B) along the b direction; (**b**) distribution of the corresponding diffusion energy barriers of Ca^2+^ at the VO_2_ (B)/rGO interface along the a direction. Reproduced with permission [73]. Copyright 2024, Wiley-VCH GmbH. (**c**) Li_2_S decomposition energy barriers on the CB, VO_2_, and Fe-VO_2_ surfaces. (**d**) Rate performance of CB, VO_2_@CB, and Fe-VO_2_@CB electrodes from 0.1 to 4 C. Reproduced with permission [74]. Copyright 2023, Wiley-VCH GmbH. DOS and PDOS of (**e**) VO_2_ and (**f**) Cu_1mmol_-VO_2_. Reproduced with permission [75]. Copyright 2025, Wiley-VCH GmbH. (**g**) Area capacitance of VO_2_ and Co-VO_2_ anodes at 1–20 mA cm^−2^. (**h**) GCD curves of A-HSC. Reproduced with permission [76]. Copyright 2024, American Chemical Society. (**i**) Ragone plot of a VO_2_/V_2_C MXene-based asymmetric supercapacitor. Reproduced with permission [77]. Copyright 2024, Elsevier.

CIBs have garnered interest because of their low redox potential, abundant calcium resources, and low propensity for dendrite formation. Wang et al. fabricated a VO_2_ (B)/rGO heterojunction cathode in which VO_2_ (B) offered large tunnels for Ca^2+^ diffusion [73], while the V-O-C bonding between VO_2_ (B) and rGO formed a conductive network (Figure 6a), enhancing the electronic conductivity and reducing the charge transfer resistance. The interfacial Ca^2+^ diffusion barrier was only 0.64 eV (Figure 6b), and the discharge capacity improved from 157.2 to 319.2 mAh g^−1^. Even after 3000 cycles at 50 °C, 85% of the capacity was retained, demonstrating excellent thermal stability. In lithium–sulfur batteries, VO_2_ has also been proven capable of regulating LiPS intermediate behavior. Pang et al. synthesized atomically dispersed Fe^3+^-doped VO_2_ nanoribbons (Fe-VO_2_) via a hydrothermal method [74]. Through electronic metal–support interactions (EMSIs), the electronic structure was modulated, increasing the VO_2_ conductivity by approximately three orders of magnitude. This material significantly reduced the decomposition barrier of Li_2_S from 1.60 to 1.32 eV and the Li^+^ diffusion barrier from 1.42 to 0.99 eV (Figure 6c), enabling strong LiPS adsorption and rapid conversion. The material achieved an initial capacity of 1275 mAh g^−1^ at 0.1 C and maintained 67% of its capacity after 500 cycles at 1 C, offering a new EMSI-based design approach for Li-S battery cathysts (Figure 6d). For AIBs, Wang et al. fabricated Cu-doped VO_2_ nanoflowers via a glucose-assisted hydrothermal method [75]. Cu^2+^ doping induced hybridization between the 3d orbitals and V-O orbitals, which enhanced electron coupling and narrowed the band gap from 1.18 eV to nearly zero (Figure 6e,f), improving the electronic conductivity. The material demonstrated an optimized Al^3+^ adsorption energy of −1.23 eV and a diffusion coefficient of 10–11 cm^2^ s^−1^. By tuning the oxygen vacancies, its pseudocapacitive behavior was enhanced. The Cu-VO_2_ electrode delivered an initial capacity of 642 mAh g^−1^ at 0.4 A g^−1^ and maintained 116 mAh g^−1^ after 200 cycles, exhibiting excellent full-cell performance when paired with a 5 M Al(OTF)_3_ electrolyte and an ionic liquid anode.

VO_2_ has also shown broad applicability in supercapacitors. Chen et al. introduced a Co^2+^ preinsertion strategy to stabilize the VO_2_ tunnel structure, significantly inhibiting vanadium dissolution and enhancing both electronic and structural stability [76]. The NH_4_^+^ storage mechanism was found to involve reversible intercalation and hydrogen bonding. The resulting Co-VO_2_ electrode achieved an areal capacitance of 9.5 F cm^−2^ at 1 mA cm^−2^ (Figure 6g) and retained 77.4% capacity after 2000 cycles, outperforming pristine VO_2_. A hybrid device based on Co-VO_2_/CuFe-PBA achieved an areal capacitance of 3035.8 mF cm^−2^ with stable cycling (Figure 6h). VO_2_/V_2_C MXene composites were further engineered by Zhu et al., who reported enhanced electrical conductivity and a high density of redox-active sites as a result of synergistic interfacial effects [77]. An asymmetric supercapacitor based on this composite delivered an energy density of 10.56 Wh L^−1^ at a power density of 127.8 W L^−1^ (Figure 6i), with a capacity retention of 74.2% after 5000 cycles, offering a novel strategy for the development of high-performance electrodes.

Overall, ongoing research on VO_2_ across various energy storage technologies continues to expand its material boundaries and provides critical insights and material support for designing high-performance and highly stable next-generation energy storage systems.

## 4. Summary and Outlook

Owing to its unique semiconductor properties, temperature-sensitive phase transition behavior, and highly tunable crystal structure, VO_2_ offers a crucial material foundation for the development of high-performance energy storage devices and demonstrates multidimensional application potential. In this paper, we systematically review the crystal structure of VO_2_, its mainstream synthesis methods, and the latest research progress in the field of energy storage. Currently, VO_2_ has been widely applied in lithium-ion batteries, zinc-ion batteries, photoassisted batteries, and supercapacitors. Its conductivity and structural stability have been effectively enhanced through elemental doping and composite modification. However, several challenges remain in its practical applications, including structural degradation during charge and discharge cycles, high costs associated with large-scale production, and an unclear correlation between the phase transition process and energy storage mechanisms.

In the future, the development of VO_2_ in energy storage systems may achieve breakthroughs in the following aspects:

1. Material design:

Conventional trial-and-error methods for tuning VO_2_ crystal structures are often inefficient and costly, making them inadequate for meeting the increasing demand for rapid performance optimization in energy storage applications. In contrast, a machine learning-driven design strategy integrated with high-throughput computational and experimental datasets offers a way to analyze the complex correlations between structural parameters and electrochemical performance in detail. Such theoretical models enable the rapid screening of critical factors, including dopant species, concentrations, and growth conditions, thereby facilitating the efficient optimization of material design. Through iterative model refinement, this data-driven approach can continuously generate novel VO_2_ structural configurations, significantly reduce the research and development cycle, and accelerate the paradigm shift from empirical to predictive material design.

2. Electrode construction:

For electrode structure engineering, nanoscale array architectures can be designed to optimize the exposure of active crystal facets and improve the utilization of electrochemical reaction sites; alternatively, 3D porous electrode structures can be fabricated to increase interfacial contact and promote ion transport efficiency, as exemplified by the design of 3D bionanostructures, which represents a promising direction for significant innovation.

3. Research methodology:

Characterization technologies are essential tools for investigating energy storage materials, as they reveal reaction mechanisms and structural evolution. By employing advanced in situ and synchrotron radiation-based techniques, it is possible to dynamically track phase transitions, morphological changes, and interfacial reactions during the energy storage process, thereby enabling a deeper understanding of the intrinsic relationship between structure and performance. For example, in situ X-ray diffraction can monitor real-time crystal phase transitions of electrode materials during charge–discharge cycles; in situ transmission electron microscopy enables a direct visualization of structural and morphological evolution at the nanoscale; and in situ Raman spectroscopy can track chemical bond changes associated with phase transformations. Additionally, synchrotron-based X-ray absorption spectroscopy, including both the X-ray absorption near-edge structure and extended X-ray absorption fine structure, offers precise insights into the valence states and local coordination environments of transition metals, providing critical support for understanding the electronic structure evolution of materials under dynamic electrochemical conditions. The synergistic application of these techniques facilitates a comprehensive elucidation of the coupling mechanisms between structural changes and electrochemical performance, offering a robust theoretical and data-driven foundation for the compositional design and process optimization of energy storage materials.

4. Industrialization:

The development of low-cost, environmentally friendly, scalable, and robust synthesis methods is essential for advancing the practical application of VO_2_ materials in secure and intelligent energy storage systems. Such methods ensure consistent material quality during large-scale production, while optimizing synthesis routes and adopting green technologies can further reduce costs and increase the commercialization potential of VO_2_-based energy storage devices.

## Figures and Tables

**Table 1:** Comparative performance of VO_2_ and representative electrode materials.

Materials	Cost	Capacity (mA h g^−1^)	Stability (Cycles)	Scalability	Typical Applications	Ref.
VO_2_	Moderate	400.2 (0.5 A g^−1^)	84.3% (5 A g^−1^, 6000)	Moderate	Zn-ion, Li-ion, photoassisted batteries	[37]
V_2_O_5_	Low	319 (0.02 A g^−1^)	81% (2 A g^−1^, 500)	High	Zn-ion, Li-ion, photoassisted batteries	[38]
V_6_O_13_	Moderate	394.2 (0.1 A g^−1^)	94% (2 A g^−1^, 100)	Moderate	Zn-ion, Li-ion batteries	[39]
Ti_3_C_2_I_2_	High	181 (0.25 A g^−1^)	80% (4 A g^−1^, 700)	Low	Supercapacitors, Li-ion, Na-ion batteries	[40]
MoS_2_	Moderate	156 (0.1 A g^−1^)	97.3% (1 A g^−1^, 500)	Moderate	Li-ion, Na-ion, Zn-ion batteries	[41]

Note. Since VO_2_ has been more extensively studied in the context of zinc-ion batteries, the capacity values listed in this table refer to its performance in zinc-ion battery systems for consistency in comparison.
